# Bibliometric analysis of postoperative atrial fibrillation following coronary artery bypass grafting surgery

**DOI:** 10.1097/MD.0000000000041773

**Published:** 2026-05-15

**Authors:** Taiwei Wang, Lin Luo, Xuemiao Huang, Chaoyue Gao, Yue Shu, Zexinyao Ren, Yisi Liu

**Affiliations:** a School of Nursing, Capital Medical University, Beijing, China; b Department of Cardiology, Peking Union Medical College Hospital, Chinese Academy of Medical Sciences & Peking Union Medical College, Beijing, China.

**Keywords:** bibliometric analysis, CiteSpace, coronary artery bypass grafting, postoperative atrial fibrillation, VOSviewer

## Abstract

**Background:**

Postoperative atrial fibrillation (POAF) is a common and serious complication following cardiac surgery, particularly after coronary artery bypass grafting. Despite extensive research into its pathophysiology and management, the incidence of POAF remains high, reflecting the complexity of its underlying mechanisms.

**Methods:**

This study provides a comprehensive bibliometric analysis of the literature on POAF following coronary artery bypass grafting from 1999 to 2023, utilizing tools such as VOSviewer, Bibliometrix, and CiteSpace.

**Results:**

We analyzed 1457 publications, identifying trends in publications, citation patterns, and collaborative networks. Key contributors, including influential authors, institutions, and countries, were mapped, and the thematic evolution of research was explored. Our findings highlight the prominent role of the United States in POAF research, the impact of key journals, and the evolving focus towards understanding inflammation, autonomic modulation, and epicardial adipose tissue in POAF development.

**Conclusion:**

This analysis underscores the need for further research to develop personalized prevention and management strategies, ultimately aiming to reduce the burden of POAF and improve patient outcomes.

## 1. Introduction

Postoperative atrial fibrillation (POAF), atrial fibrillation occurring after surgery, stands as a significant challenge in the realm of surgical medicine, particularly within the domain of cardiac surgery.^[1]^ POAF’s deleterious effects, including prolonged hospital stays, heightened healthcare costs, and increased long-term risks, underscore the urgency for a deeper understanding of this complication.^[2]^ While its association with adverse cardiovascular events like stroke, heart failure, and mortality after both cardiac and non-cardiac surgeries is well-documented,^[3–6]^ the precise causal mechanisms warrant further elucidation. Its prevalence, coupled with its adverse impact on patient outcomes, underscores the importance of continued research into its etiology, management, and prevention strategies.

The incidence of POAF varies across different surgical procedures, with cardiac surgeries exhibiting higher rates compared to non-cardiac procedures.^[7]^ Post-cardiac surgery, the overall incidence of POAF is approximately 30%, with a higher occurrence rate in valve surgeries, reaching up to 40% to 50%.^[8]^ Notably, despite widespread recognition of its impact on clinical outcomes and the abundance of research into both novel and established preventative strategies, the incidence of POAF has remained relatively stable over the decades.^[2,9]^

The persistent nature of POAF despite decades of research underscores the complexity of its pathophysiology. The genesis of POAF is attributed to a constellation of unique conditions including direct impair to the myocardium,^[7]^ surgery-related inflammatory responses,^[10,11]^ metabolic activity of peri-atrial adipose tissue,^[12,13]^ and autonomic neuromodulation.^[14]^

Efforts to prevent POAF have encompassed a range of pharmacological and procedural interventions, albeit with varying degrees of success. β-blockers and amiodarone stand out as effective agents in mitigating its occurrence,^[15]^ while strategies like pericardiotomy^[16]^ and anti-inflammatory medications^[17]^ show promise but require further validation.

Bibliometrics, a quantitative method used to analyze the literature and evaluate trends in research activities. Utilizing multiple bibliometric analysis tools, we conduct a comprehensive bibliometric analysis on POAF following coronary artery bypass graft (CABG) surgery research to discern publication trends, citation patterns, and thematic evolution. Our objectives include identifying influential authors, institutions, and countries, mapping collaboration networks, and uncovering prevalent topics and emerging trends. Although publications present a general overview of POAF, no bibliometric analysis of this field has been conducted. This study fills a gap in the literature by offering insights into POAF after CABG research from 1900 to 2023, serving as a valuable resource for clinicians, researchers, and policymakers involved in cardiac surgery patient management.

## 2. Methods

### 2.1. Data acquisition

**Figure 1.**
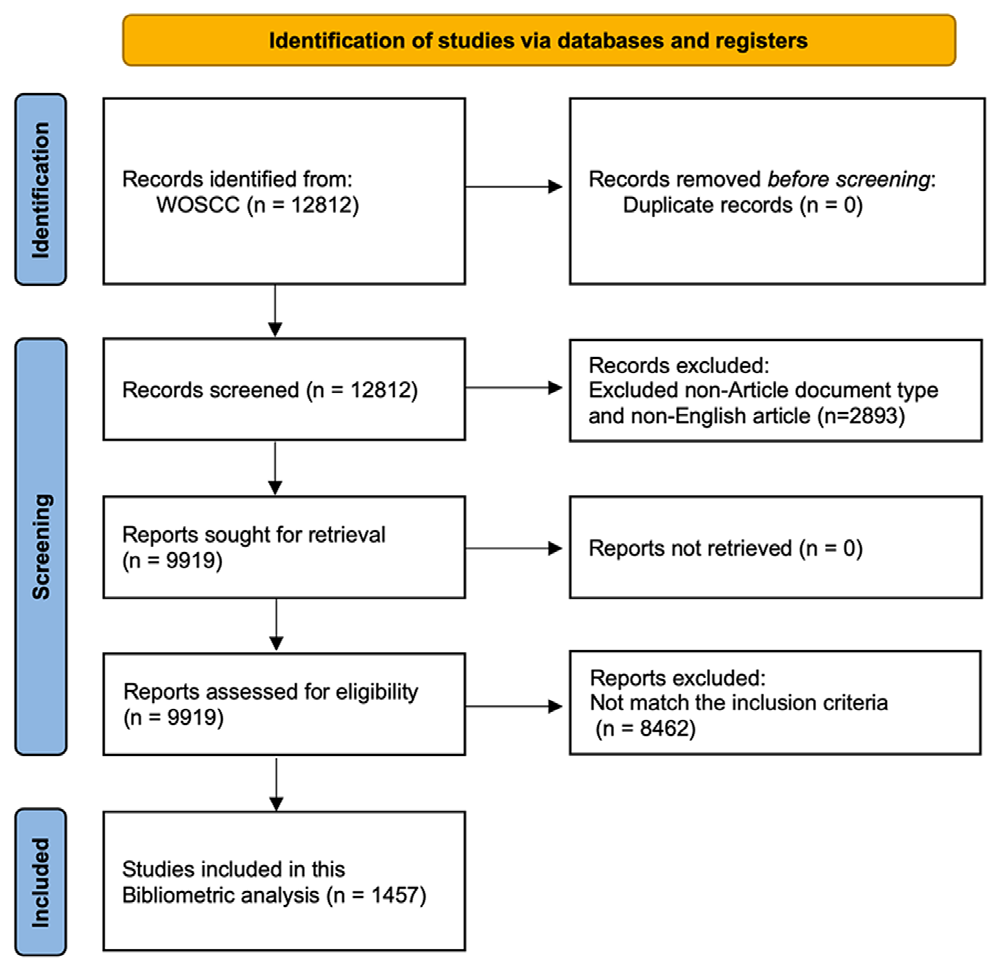
Flowchart of the literature search.

The scientific literature was meticulously curated from the Web of Science (WoS) Core Collection database as of December 15, 2023. Our comprehensive search strategy is elaborated in Supplementary Table 1 (STable1). In order to uphold the integrity of our analysis and preempt any potential biases stemming from database updates, all data extraction and downloading processes were diligently completed on the same date. Initially, our search yielded a total of 12,812 publications, which underwent further scrutiny through examination of titles and abstracts. This meticulous review enabled us to pinpoint articles that closely aligned with the objectives of our study. Subsequently, following this rigorous screening process, a subset of 1457 publications was deemed suitable for inclusion in our bibliometric analysis (Fig. 1). The search results were exported in “Plain Text file” format with the record content set to “Full Record and Cited References,” and stored in download_*.txt format for subsequent analysis.

### 2.2. Bibliometric Analysis and Visualization

In this study, data extracted from the available literature were meticulously analyzed and visualized using several software tools. Microsoft Excel (version 2021), VOSviewer (version 1.6.20), Bibliometrix (version R.4.1.4), and Citespace (version 6.1.6) were employed for this purpose.

Initially, the raw data downloaded from WoS were imported into Microsoft Office Excel (Microsoft Corporation, Redmond, WA, United States) for preliminary collation of bibliometric indicators. These indicators encompassed various metrics such as the quantity of papers, frequency of citations, H-index, leading countries, institutions, journals, and authors.

VOSviewer,^[18]^ a powerful visualization tool developed by Leiden University, was utilized to analyze and visualize the co-occurrence network of keywords, intercountry, and inter-institution collaboration axes. This software offers robust capabilities in calculating and visualizing complex networks.

R Studio (R 4.3.2) facilitated advanced bibliometric analysis, with the Bibliometrix R package^[19]^ being employed specifically for constructing WordCloud maps following package tutorials. Additionally, the distribution of publications across the world was illustrated using “ggplot2” and “rworldmap” packages.

CiteSpace,^[20]^ developed by Prof Chaomei Chen’s team, was employed for metrological analysis visualization, particularly for co-citation analysis and citation burst evaluation. Citespace was configured with specific parameter settings including a time span from Jan. 1999 to Dec. 2023, node type set to reference, and pruning methods applied to “Pathfinder/pruning sliced network/pruning the merged network”

### 2.3. Ethical considerations

As this study did not involve human participants or animal subjects, ethical approval was not required.

## 3. Results

### 3.1. Descriptive analysis of publications

**Figure 2.**
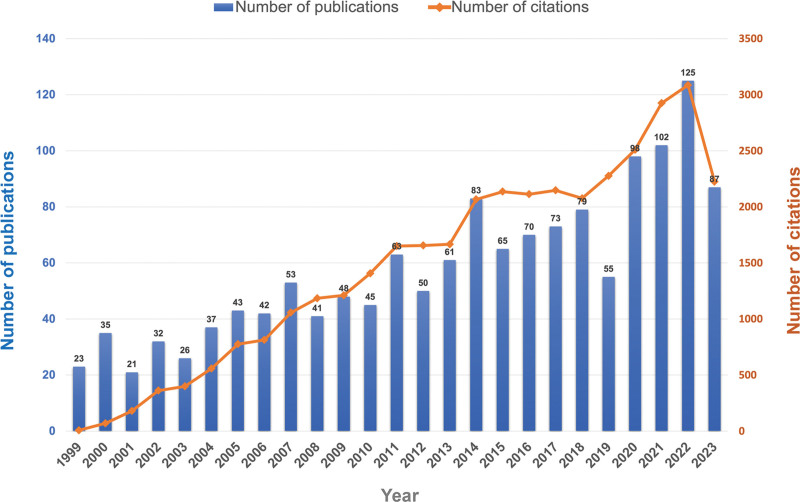
Trend of the numbers of publications and citations from 1999 to 2023.

The distribution of the number of publications and citations in the field of POAF from 1999 to 2023 by year (Fig. 2). With the increasing attention of researchers to the field of POAF in recent years, the number of publications per year increased steadily. Moreover, the number of citations shown the same upward trend. In 2022, the field experienced its peak, marked by a significant milestone: 125 publications and 3086 citations, the highest figures recorded throughout the analyzed period.

A total of 1457 enrolled publications have received a cumulative total of 36562 citations, with 27146 excluding self-citations. The average citation frequency per article is 25.1, showcasing the substantial impact of POAF research. The H-index stands at 85, indicating significant scholarly influence. The top 100 most cited articles contribute 15,406 citations (42.13% of the total), with each article cited approximately 154.06 times. The top 50 articles receive 10,687 citations (29.23% of the total), with an average citation frequency of 213.74 times per article. These findings underscore the prominence and impact of top-cited POAF literature.

### 3.2. Publications distribution among nations

**Figure 3.**
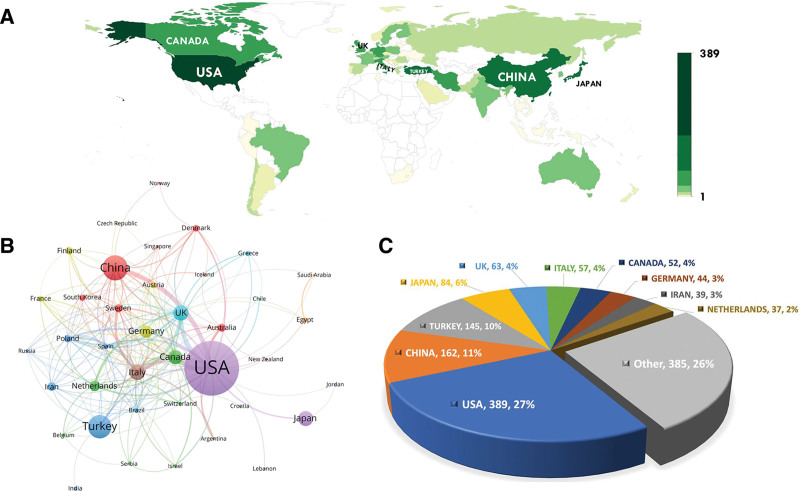
Global research on POAF. (A) Comparative map of the distribution of the total number of publications among nations, and (C) the top 10 most productive countries. (B) Visualization of cooperation networks among nations. POAF = postoperative atrial fibrillation.

The eligible publications were published in 59 countries among nations. The top 10 most productive nations were shown in pie chat in Figure 3C, which the USA rank the first, followed by China, Turkey, Japan and UK. Comparative map of the distribution of the total number of publications among nations was generated by “ggplot2” and “rworldmap” packages (Fig. 3A).

In the visualization of cooperation networks among nations, the number of links reflects international collaborations, with more lines indicating greater activity. Additionally, the thickness of the lines between countries signifies the strength of their collaborative relationship, with thicker lines indicating stronger cooperation. As referential in Figure 3B, the USA has the most interconnected targets and link strength in terms of international cooperation. Besides, the top 10 productive countries were also rank in the top of international cooperation.

### 3.3. Publications distribution across institutions

**Figure 4.**
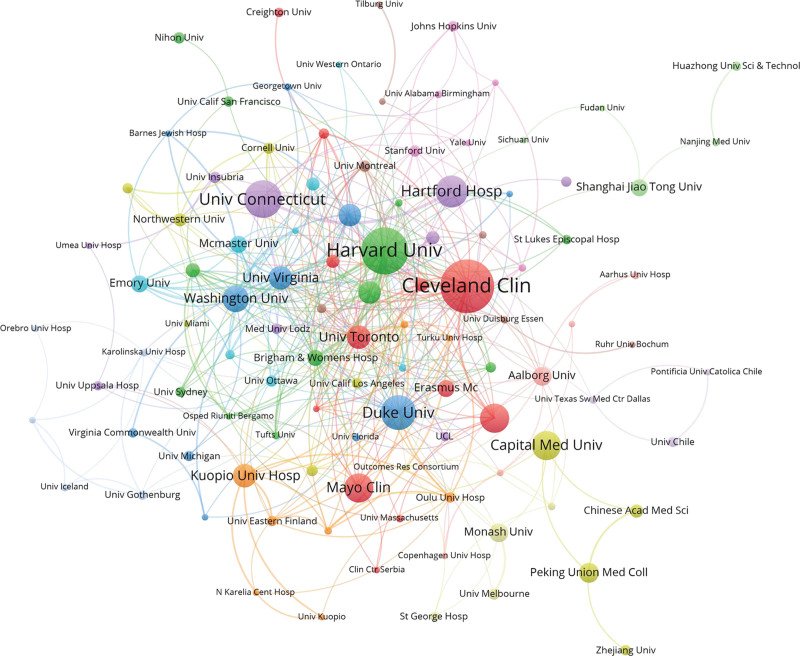
Visualization of cooperation networks across institutions.

A total of 128 institutions involved in the investigation of the field of POAF. The first 10 most yielding institutions are listed in Table 1. Interestingly, among the top 10 most prolific institutions, 8 were from the USA. Although the Cleveland Clinic published the most articles (n = 40) and most citations (n = 1739), the Duke University had the highest citations per article (n = 63.54). Capital Medical University rank the 6^th^ out of top 10, and the first among China. Among the visualization of cooperation networks across institutions (Fig. 4), the showcases clusters based on co-citation relationships among these institutions, with varying colors representing distinct co-citation associations. Institutions from the same country cooperate with each other, but international cooperation still needs to be strengthened.

**Table 1:** Top 10 institutions.

RANK	Institution	Country	Articles	Total citations	Citations per article
1	Cleveland Clinic	USA	40	1739	43.48
2	Harvard University	USA	35	1384	39.54
3	University of Connecticut	USA	28	913	32.61
4	Duke University	USA	26	1652	63.54
5	Hartford Hospital	USA	24	766	31.92
6	Capital Medical University	China	22	113	5.14
6	Case Western Reserve University	USA	22	787	35.77
6	Mayo Clinic	USA	22	889	40.41
9	Washington University	USA	20	984	49.20
10	Kuopio University Hospital	Finland	18	498	27.67

### 3.4. Analysis of high-contributing journals

**Figure 5.**
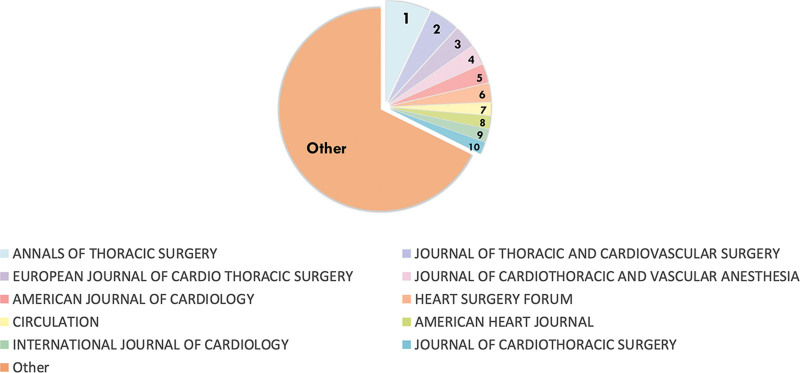
Visualization of top 10 high-contributing journals.

During the time span from 1999 to 2023, 360 scientific journals had published all this 1457 articles. The top 10 high-contributing journals in terms of publication volume related to the topic of POAF are presented in Figure 5. Using 5 main indexes, including total article, total citations, citations per article, journal impact factor, and JCR quartile, the information of these 10 journals were list in Table 2. The top 10 journals published a total of 466 papers (31.98%). Among them, ANNALS OF THORACIC SURGERY had the highest number of publications (n = 102), and the highest total citations (n = 1495). It’s noteworthy that CIRCULATION, ranking 8th, has published 30 articles related to POAF, each garnering an impressive average of 132.1 citations per article, far surpassing others in the top 10. This indicates that researchers are paying significant attention to the papers published in the journal. Additionally, 5 journals were categorized within the JCR Q1 division, signifying their esteemed academic status. Remarkably, 8 of these journals hail from the USA, while the remaining 2 originate from the Netherlands and the UK. As all these nations are developed, they collectively offer pivotal platforms for advancing research in this field.

**Table 2:** Top 10 journals.

RANK	Journal title	Articles (%)		Total citations	Citations per article	IF-2022	JCR	Country
1	ANNALS OF THORACIC SURGERY	102	7.00	4195	41.13	4.6	Q1	USA
2	JOURNAL OF THORACIC AND CARDIOVASCULAR SURGERY	68	4.67	2210	32.50	6.0	Q1	USA
3	EUROPEAN JOURNAL OF CARDIO-THORACIC SURGERY	51	3.50	1412	27.69	3.4	Q1	Netherlands
4	JOURNAL OF CARDIOTHORACIC AND VASCULAR ANESTHESIA	44	3.02	709	16.11	2.8	Q3	USA
5	AMERICAN JOURNAL OF CARDIOLOGY	43	2.95	1479	34.40	2.8	Q3	USA
6	HEART SURGERY FORUM	42	2.88	335	7.98	0.4	Q4	USA
7	CIRCULATION	30	2.06	3963	132.10	37.8	Q1	USA
8	AMERICAN HEART JOURNAL	29	1.99	1139	39.28	37.8	Q1	USA
9	INTERNATIONAL JOURNAL OF CARDIOLOGY	29	1.99	554	19.10	4.8	Q2	Ireland
10	JOURNAL OF CARDIOTHORACIC SURGERY	28	1.92	353	12.61	1.6	Q3	UK

### 3.5. Analysis of first authors and corresponding authors

The top 10 most productive first-authors in this field are shown in Table 3. Due to the prevalence of an equal number of authors, ultimately, there are 20 authors listed. All these authors published 94 articles, accounting for 6.45%.

**Table 3:** Top 10 first authors.

Rank	Corresponding author	Country	Institution	TP	TC	TC/TP	HCP	TFP
1	Mariscalco, Giovanni	USA	University of Leicester	9	699	77.67	237	2006
2	Filardo, Giovanni	USA	Baylor Scott & White Health	7	303	43.29	96	2009
3	Sezai, Akira	Japan	Nihon University	6	226	37.67	72	2009
4	Hakala, Tapio	Finland	Kuopio University Hospital	5	98	19.60	40	2002
4	Amar, David	USA	Memorial Sloan-Kettering Cancer Cente	5	482	96.40	156	1999
4	Banach, Maciej	Poland	Medical University of Lodz	5	253	50.60	107	2006
4	Rader, Florian	USA	Cedars Sinai Medical Center	5	85	17.00	28	2010
8	Baker, William L.	USA	University of Connecticut	4	106	26.50	45	2007
8	Benedetto, Umberto	UK	University of Bristol	4	114	28.50	44	2007
8	Bramer, Sander	Netherlands	Catharina Hospital	4	138	34.50	73	2010
8	Efird, Jimmy T.	Australia	University of Newcastle	4	18	4.50	9	2014
8	Gillespie, Effie L.	USA	University of Connecticut	4	55	13.75	25	2005
8	Kertai, Miklos D.	USA	Duke University	4	65	16.25	24	2014
8	Kinoshita, Takeshi	Japan	Shiga University of Medical Science	4	94	23.50	46	2010
8	Melduni, Rowlens M.	USA	Mayo Clinic	4	223	55.75	91	2011
8	Ozaydin, Mehmet	Turkey	Suleyman Demirel University	4	210	52.50	115	2008
8	Saxena, Akshat	Australia	St. Vincent’s Hospital Melbourne	4	189	47.25	119	2012
8	Sigurdsson, Martin I.	USA	Harvard Medical School	4	41	10.25	17	2015
8	Thoren, Emma	Sweden	Uppsala University	4	113	28.25	37	2012
8	Zhang, Jian	China	General Hospital of Northern Theater Command	4	51	12.75	26	2017

HCP = highest cited publication, TC = total citations, TFP = time of first publication, TP = total publications.

Giovanni Mariscalco from University of Leicester, who was previously affiliated with the University of Leicester, has made the most significant contributions to POAF research (n = 9). Giovanni M’s research focus on examines factors influencing POAF after cardiac surgery, including predictive tools like the POAF score^[21]^ and interventions such as preoperative n-3 polyunsaturated fatty acids.^[22]^ Giovanni M’s highest cited publication (n = 237), also the highest among all the top 10 authors, investigate the relationship between POAF and long-term outcomes, aiming to improve clinical management strategies.^[23]^ Considering the timing of their initial publications, Amar, David^[24]^ is among the earliest researchers in the field, with his first articles dating back to 1999.

Additionally, the top 10 corresponding authors with most publications in this field are shown in Table 4, a total of 17 authors were listed. All these authors published 111 articles, accounting for 7.62%.

**Table 4:** Top 10 corresponding authors.

Rank	Corresponding author	Country	Institution	TP	TC	TC/TP	HCP	TFP
1	Coleman, Craig I.	USA	University of Connecticut	12	333	28	61	2004
2	Mariscalco, Giovanni	USA	University of Leicester	9	699	78	237	2006
3	de Groot, Natasja M. S.	Netherlands	Erasmus University Medical Center	8	55	7	20	2017
3	Filardo, Giovanni	USA	Baylor Scott & White Health	8	328	41	96	2009
4	Gaudino, Mario	USA	Weill Cornell Medicine	7	414	59	349	2003
4	Sezai, Akira	Japan	Nihon University	7	238	34	71	2009
4	Wang, Huishan	China	General Hospital of Northern Theater Command	7	90	13	26	2017
8	Wang, Shuiyun	China	Fuwai Hospital	7	32	5	13	2017
9	Amar, David	USA	Memorial Sloan Kettering Cancer Center	6	513	86	156	1999
10	Auer, Johann	Austria	General Hospital Wels	5	362	72	105	2004
10	Banach, Maciej	Poland	Medical University Lodz	5	253	51	107	2006
10	Engin, M.	Turkey	University of Health Sciences Turkey	5	35	7	14	2020
10	Hakala, Tapio	Finland	Kuopio University Hospital	5	98	20	40	2002
10	Jahangiri, Marjan	UK	University of London	5	222	44	85	2005
10	Niessner, Alexander	Austria	Medical University of Vienna	5	27	5	16	2018
10	Ozsin, Kadir Kaan	Turkey	University of Health Sciences Turkey	5	39	8	20	2018
10	Wu, Ying	China	Capital Medical University	5	28	6	15	2018

HCP = highest cited publication, TC = total citations, TFP = time of first publication, TP = total publications.

Coleman, Craig I. from University of Connecticut lead this list in terms of publications (n = 12), followed by Mariscalco, Giovanni (n = 9), de Groot, Natasja M. S. (n = 8), and Filardo, Giovanni (n = 8). Three researchers from China have been included in this list, namely Wang, Huishan (General Hospital of Northern Theater Command, n = 7), Wang, Shuiyun (Fuwai Hospital, n = 7), and Wu, Ying (Capital Medical University, n = 5). A study researched by Mario, Gaudino has garnered the highest citation count among all publications by authors listed in the ranking.^[25]^ In this study, the -174G/C Interleukin-6 promoter gene variant was found to modulate the inflammatory response to surgery and influence the development of postoperative atrial fibrillation, suggesting both an inflammatory component of postoperative atrial arrhythmias and a genetic predisposition to this complication.

### 3.6. Analysis of articles citations

The analysis of citations, conducted using WoSCC-SCIE, serves as a dependable metric for evaluating the caliber of scholarly articles.^[26]^ STable 2 displays the top 50 most cited articles, providing insights into their impact and significance within the field.

The article published, corresponding authored by Mathew JP, in JAMA in 2004 ranked first.^[27]^ This study examined the incidence and impacts of atrial fibrillation following CABG surgery, identifying key risk factors and developing predictive models.^[27]^ Results underscore the significance of postoperative interventions like beta-blockers and ACE inhibitors in reducing atrial fibrillation occurrence and associated complications, emphasizing the importance of targeted preventative strategies in CABG patients.^[27]^ A trial published in *Circulation* in 2006 ranked the second.^[28]^ This research demonstrated that preoperative treatment with atorvastatin significantly reduces the incidence of PAOF and shortens hospital stay in patients undergoing elective cardiac surgery with cardiopulmonary bypass.^[28]^ The third one investigates the impact of PAOF on mortality following CABG, revealing that patients experiencing POAF exhibit increased in-hospital mortality, strokes, and longer hospital stays, with worse long-term survival compared to those without AF.^[29]^ The fourth^[30]^ and fifth^[25]^ studies confirm a robust correlation between inflammation and oxidative stress in the initiation of POAF, respectively. Among the top 50 most cited articles, 12 were from *Circulation*, 8 were from *Journal of the American College of Cardiology,* and 7 were from *Annals of thoracic surgery*. In addition, among the top 10 most cited articles, 4 were from *Circulation*, 2 were from *Journal of the American College of Cardiology*, and 2 were from *JAMA*. It is worth noting that most of the above 3 journals were also listed in the top 10 journals list. This table (STable 2) compiles the top 50 influential articles on POAF, considered as classics in the field. Studying these articles holds significant importance for gaining deeper insights into key research directions in the realm of POAF.

### 3.7. Analysis of keywords

Keywords are crucial for analyzing data sources as they unveil the primary contents of existing research.^[31]^ They summarize information regarding terms, objectives, methodologies, and themes of articles. Keyword co-occurrence occurs when 2 or more keywords appear together in the same article, facilitating the identification of hot topics and tracking transitions in research frontiers within the scientific domain.^[32]^ Keywords undergo preprocessing to account for variations in the same word, thereby ensuring unbiased expressions in publications. For instance, variations of the term “ CABG “ such as “ coronary artery bypass graft surgery” and “ coronary artery bypass grafting surgery” are employed to maintain consistency in analysis.

**Figure 6.**
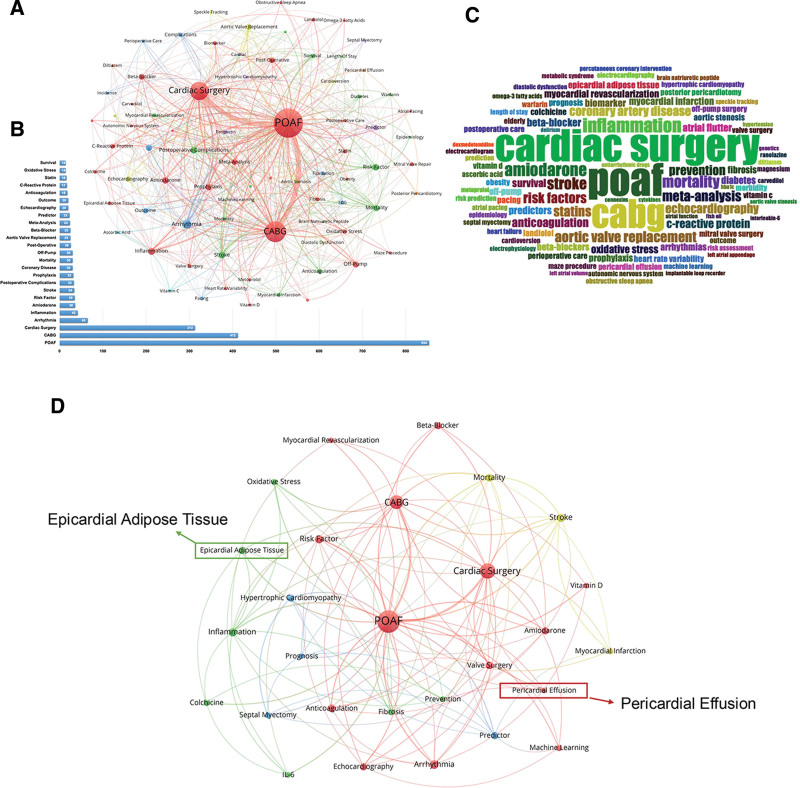
Visualization of keyword analysis. (A) VOSviewer visualization map of keywords analysis. (B) Top 25 most frequently used keywords. (C) Bibliometrix keyword WordCloud map. (D) VOSviewer visualization map of keywords analysis within 5 years

To perform our analysis, we extracted authors keywords from the eligible articles, and keywords with an occurrence of > 5 were entered into the final analysis. All terminologies were selected by the researchers, and a network visualization graph is presented in Figure 6A. A total of 78 authors keywords were extracted, among which 3 words appeared > 100 times and 13 words appeared > 30 times. The top 25 most frequently used keywords are shown in Figure 6B. A keyword WordCloud map, constructed by Bibliometrix R package, was shown in Figure 6C. The keyword “CABG” was rank the second in this list, which indicated that CABG surgery is the most widely studied cardiac surgery in the field of postoperative atrial fibrillation. In the top keyword list, following the prominent terms POAF, CABG, cardiac surgery, and arrhythmia, the subsequent rankings include inflammation, amiodarone, risk factor, and stroke. These are all highly researched directions within the field of POAF.

Furthermore, under the same screening conditions, we analyzed the top keywords within 5 years (Fig. 6D). The 2 keywords highlighted with arrows and boxes, “Epicardial Adipose Tissue” and “Pericardial Effusion,” are notable areas of interest. Epicardial adipose tissue is closely associated with cardiac inflammatory responses^[33]^ and may play a critical role in the onset and progression of postoperative atrial fibrillation, making it a recent focus of research. Meanwhile, pericardial effusion, as a common postoperative complication, reflects the local cardiac environment after surgery, has consequently drawn significant attention. The emphasis on these 2 keywords indicates their significance and potential in the current field of POAF research, warranting further investigation to better understand their pathophysiological mechanisms and clinical implications in postoperative atrial fibrillation.

### 3.8. Analysis of citations burst

**Figure 7.**
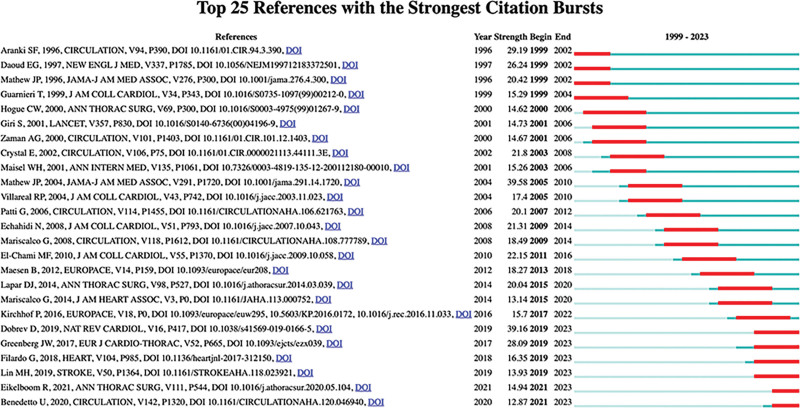
Top 25 references with the strongest citation bursts.

In the realm of bibliometrics, references undergoing citation bursts, frequently cited by other studies within specific time frames, serve as pivotal indicators of recognition and influence within the academic sphere.^[34]^ The significance of such bursts lies in their capacity to pinpoint research hotspots, seminal papers, and track the trajectory of research themes.^[35]^ Analyzing 1457 articles and their corresponding 18392 references sourced from WoSCC-SCIE between 1999 and 2023 using CiteSpace, we delved into the robust citation bursts within this domain. The references from the top 25 entries exhibiting the most pronounced citation bursts are illustrated in Figure 7, while their respective titles are delineated in STable 3. “Year” denotes the publication date, “Begin” signifies the initial citation, and “End” denotes the final citation within the specified period. The reference with most strength was a review titled “Postoperative atrial fibrillation: mechanisms, manifestations and management” by Dobrev D in 2019.^[7]^ It is noteworthy that this publication continues to experience citation bursts up to the present, indicating its recent and sustained attention within the academic community. Furthermore, Mathew JP and Mariscalco G each have 2 publications listed in the top 25 citation burst rankings, and the articles published by Mathew JP are dated earlier.

## 4. Discussion

### 4.1. General information

This bibliometric analysis scrutinized 1457 articles on postoperative atrial fibrillation from 1999 to 2023 in the WoSCC-SCIE. Using VOSviewer, CiteSpace, and Bibliometrix software, we examined publication trends to offer valuable insights for researchers in this field.

The upward trend in both the number of publications and citations in the field of POAF reflects the growing attention and interest of researchers in recent years. This surge in research output suggests an increasing recognition of the significance of POAF in clinical practice and academia. The peak in 2022, marked by 125 publications and 3086 citations, indicating a period of heightened activity and scholarly engagement recently.

The analysis of publication distribution among nations reveals a diverse landscape of research contributions to the field of POAF. The United States emerges as the most prolific nation, demonstrating a robust commitment to POAF research. This dominance is followed by significant contributions from China, Turkey, Japan, and the United Kingdom, showcasing a global interest in addressing POAF-related challenges. The comparative map of publication distribution among nations provides a visual representation of the geographic spread of research activity, highlighting the international scope of POAF research endeavors. The visualization of cooperation networks among nations elucidates the dynamics of international collaboration in POAF research. The United States emerges as a central hub in the cooperation network, indicating its pivotal role in facilitating collaborative efforts across borders. The strong interconnectedness and link strength associated with the USA underscore its leadership in fostering international collaborations.

Institutions play a crucial role in driving POAF research forward, as evidenced by the analysis of publication distribution across institutions. The concentration of top-performing institutions in the United States underscores the country’s dominance in POAF research. Notably, the Cleveland Clinic emerges as a prolific contributor, publishing the highest number of articles and garnering the most citations. However, the presence of Capital Medical University among the top-ranking institutions reflects China’s growing prominence in the field. While domestic collaborations among institutions are evident, there remains untapped potential for strengthening international cooperation to enhance research outcomes and knowledge exchange.

The analysis of high-contributing journals sheds light on the scholarly dissemination of POAF research findings. Journals such as ANNALS OF THORACIC SURGERY emerge as key platforms for publishing POAF-related research, attesting to their significance in advancing knowledge in the field. The exceptional performance of journals like CIRCULATION, with its high citations per article, underscores the impact and visibility of POAF research published in these outlets. Furthermore, the predominance of journals from developed nations in the top 10 list highlights the pivotal role of these countries in shaping the scholarly discourse surrounding POAF.

### 4.2. Highly cited publications clustering

After meticulously analyzing the top 50 cited articles in the field of POAF, these studies can be classified into several primary themes, each with concise descriptions:

POAF pathophysiology: Research suggests that inflammation may play a significant role in the development of POAF. These studies investigate how inflammatory markers (e.g., C-reactive protein^[36]^), oxidative stress responses,^[37,38]^ and specific gene polymorphisms (e.g., interleukin-6 polymorphism^[25]^) influence the occurrence of POAF.

Pharmacological Prevention and Treatment: Studies investigate the efficacy of different drugs in preventing or mitigating the incidence of POAF. The investigated agents include statins, such as atorvastatin, which have shown promise in modulating lipid profiles and reducing inflammatory responses.^[39]^ Anti-inflammatory drugs, including corticosteroids^[40]^ and colchicine,^[41]^ are examined for their potential to decrease systemic inflammation that may contribute to POAF. Antiarrhythmic drugs, particularly amiodarone^[42]^ and vernakalant,^[43]^ are assessed for their direct effects on cardiac electrophysiology. Additionally, the role of Omega-3 fatty acid supplements^[44,45]^ and angiotensin-converting enzyme inhibitors^[46]^ are explored for their multifaceted cardiovascular benefits, including heart rate control and reduction of sympathetic nervous system activity.

Risk factors and risk assessment: Studies focus on identifying risk factors for POAF, such as age, obesity, metabolic syndrome, and baseline health status of patients, and develop models and scoring systems to predict POAF.^[21,27,47]^

### 4.3. Citation burst clustering

By clustering the citation burst, we can not only better understand the multifaceted impacts of POAF but also identify potential targets for prevention and treatment strategies, providing direction for future research and clinical practice.

There is a discernible evolution in research focus and direction concerning the prevention of POAF following CABG surgery. Initially, studies primarily concentrate on identifying predictive factors for POAF post-surgery, including preoperative use of amiodarone,^[48]^ alongside examining the impact of atrial fibrillation on patient outcomes and resource utilization.^[49]^

As research progresses, there is a shift towards exploring various prevention measures, such as investigating the efficacy of intravenous^[50]^ and oral amiodarone administration.^[42]^ This phase often witnesses the emergence of multicenter, randomized controlled trials aimed at validating the efficacy of pharmacological interventions in preventing POAF.

In more recent literature, the focus transitions towards examining outcomes post-POAF,^[51]^ encompassing factors like long-term survival rates^[23]^ and cardiovascular event risks. Concurrently, there is a growing exploration of the role of medications like atorvastatin in preventing POAF. Moreover, this stage sees an increase in meta-analyses and systematic reviews aimed at summarizing the findings of numerous studies. Additionally, there is a surge in studies focusing on prediction tools and risk assessment to aid decision-making in clinical practice.

Overall, the trajectory of research unfolds from identifying predictive factors and exploring prevention measures of atrial fibrillation to a deeper understanding of post-POAF outcomes and treatment strategies, with a growing emphasis on personalized prevention and management approaches.

## 5. Conclusion

This study presents a bibliometric analysis of POAF after CABG, from 1999 to 2023, with publication patterns and emerging trajectories. POAF remains a significant concern after CABG surgery due to its association with adverse outcomes such as stroke, heart failure, and increased mortality. This analysis identified key contributors, including leading countries, institutions, and authors, and elucidated the patterns of international collaboration and influential journals in this field. Despite advances in understanding the pathophysiology of POAF, including the roles of inflammation, autonomic modulation, and epicardial adipose tissue, its incidence remains stable, indicating the complexity of its underlying mechanisms and the need for more targeted prevention and management strategies.

Moving forward, there is a pressing need for continued interdisciplinary research to better understand the multifactorial etiology of POAF and to develop innovative, personalized interventions to reduce its prevalence and impact.

## Author contributions

**Conceptualization:** Taiwei Wang, Yisi Liu.

**Data curation:** Taiwei Wang, Lin Luo.

**Formal analysis:** Taiwei Wang, Lin Luo, Xuemiao Huang, Chaoyue Gao, Yue Shu.

**Funding acquisition:** Yisi Liu.

**Methodology:** Chaoyue Gao.

**Writing – original draft:** Taiwei Wang, Chaoyue Gao.

**Writing – review & editing:** Xuemiao Huang, Yue Shu, Zexinyao Ren, Yisi Liu.






