# Supplementary material for: Bibliometric analysis of postoperative atrial fibrillation following coronary artery bypass grafting surgery
Source: Medicine (Baltimore). 2026 May 15;105(20):e41773. doi: 10.1097/MD.0000000000041773 (PMC13183158; doi:10.1097/MD.0000000000041773)
Supplement: Supplementary file 1 [file medi-105-e41773-s001.docx]

**Supplementary Table 1. Search strategy**

|  | Terms | Results |
| --- | --- | --- |
| 1 | TI=(CABG OR "coronary artery bypass" OR valve OR transplantation OR stent OR coronary OR heart OR cardio OR cardiac* OR cardio* ) OR AB=(CABG OR "coronary artery bypass" OR valve OR transplantation OR stent OR coronary OR heart OR cardio OR cardiac* OR cardio* ) | 2338101 |
| 2 | TI=(surgery OR surgical OR operation OR procedure OR operative OR operative* OR postoperative OR post-operative) OR AB=(surgery OR surgical OR operation OR procedure OR operative OR operative* OR postoperative OR post-operative) | 4133101 |
| 3 | TI=("atrial fibrillation" OR "atrium fibrillation" OR "auricular fibrillation") OR AB=("atrial fibrillation" OR "atrium fibrillation" OR "auricular fibrillation") | 95708 |
| 4 | #1 AND #2 AND #3 | 12812 |
