# Supplementary material for: Bibliometric analysis of postoperative atrial fibrillation following coronary artery bypass grafting surgery
Source: Medicine (Baltimore). 2026 May 15;105(20):e41773. doi: 10.1097/MD.0000000000041773 (PMC13183158; doi:10.1097/MD.0000000000041773)
Supplement: Supplementary file 2 [file medi-105-e41773-s002.docx]

Supplementary Table 2. Top 50 most cited articles

| **Rank** | **Title** | **Journal** | **Corresponding Author** | **Year** | **TC** |
| --- | --- | --- | --- | --- | --- |
| 1 | A multicenter risk index for atrial fibrillation after cardiac surgery | JAMA-JOURNAL OF THE AMERICAN MEDICAL ASSOCIATION | MATHEW, JP | 2004 | 877 |
| 2 | Randomized trial of atorvastatin for reduction of postoperative atrial fibrillation in patients undergoing cardiac surgery - Results of the ARMYDA-3 (Atorvastatin for reduction of MYocardial dysrhythmia after cardiac surgery) study | CIRCULATION | DI SCIASCIO, G | 2006 | 501 |
| 3 | Postoperative atrial fibrillation and mortality after coronary artery bypass surgery | JOURNAL OF THE AMERICAN COLLEGE OF CARDIOLOGY | VILLAREAL, RP | 2004 | 483 |
| 4 | Ascorbate attenuates atrial pacing-induced peroxynitrite formation and electrical remodeling and decreases the incidence of postoperative atrial fibrillation | CIRCULATION RESEARCH | VAN WAGONER, DR | 2001 | 397 |
| 5 | The-174G/C interleukin-6 polymorphism influences postoperative interleukin-6 levels and postoperative atrial fibrillation. Is atrial fibrillation an inflammatory complication? | CIRCULATION | GAUDINO, M | 2003 | 349 |
| 6 | Corticosteroids for the prevention of atrial fibrillation after cardiac surgery: A randomized controlled trial | JAMA-JOURNAL OF THE AMERICAN MEDICAL ASSOCIATION | HALONEN, J | 2007 | 256 |
| 7 | Atrial fibrillation after coronary artery bypass surgery - A model for preoperative risk stratification | CIRCULATION | ZAMAN, AG | 2000 | 248 |
| 8 | Stroke after coronary artery bypass - Incidence, predictors, and clinical outcome | STROKE | STAMOU, SC | 2001 | 245 |
| 9 | New-Onset Atrial Fibrillation Predicts Long-Term Mortality After Coronary Artery Bypass Graft | JOURNAL OF THE AMERICAN COLLEGE OF CARDIOLOGY | EL-CHAMI, MF | 2010 | 242 |
| 10 | Atrial fibrillation after isolated coronary surgery affects late survival | CIRCULATION | MARISCALCO, G | 2008 | 237 |
| 11 | The HMG-CoA reductase inhibitor atorvastatin prevents atrial fibrillation by inhibiting inflammation in a canine sterile pericarditis model | CARDIOVASCULAR RESEARCH | KUMAGAI, K | 2004 | 228 |
| 12 | Colchicine for Prevention of Postpericardiotomy Syndrome and Postoperative Atrial Fibrillation The COPPS-2 Randomized Clinical Trial | JAMA-JOURNAL OF THE AMERICAN MEDICAL ASSOCIATION | IMAZIO, M | 2014 | 226 |
| 13 | Inflammation of atrium after cardiac surgery is associated with inhomogeneity of atrial conduction and atrial fibrillation | CIRCULATION | ISHII, Y | 2005 | 220 |
| 14 | Rate Control versus Rhythm Control for Atrial Fibrillation after Cardiac Surgery | NEW ENGLAND JOURNAL OF MEDICINE | GELIJNS, AC | 2016 | 216 |
| 15 | Predictors of atrial fibrillation after conventional and beating heart coronary surgery - A prospective, randomized study | CIRCULATION | ANGELINI, GD | 2000 | 216 |
| 16 | Usefulness of Neutrophil/Lymphocyte Ratio As Predictor of New-Onset Atrial Fibrillation After Coronary Artery Bypass Grafting | AMERICAN JOURNAL OF CARDIOLOGY | HILLIS, GS | 2010 | 214 |
| 17 | Colchicine Reduces Postoperative Atrial Fibrillation Results of the Colchicine for the Prevention of the Postpericardiotomy Syndrome (COPPS) Atrial Fibrillation Substudy | CIRCULATION | IMAZIO, M | 2011 | 188 |
| 18 | Postoperative Atrial Fibrillation Significantly Increases Mortality, Hospital Readmission, and Hospital Costs | ANNALS OF THORACIC SURGERY | AILAWADI, G | 2014 | 184 |
| 19 | Postoperative atrial fibrillation in patients undergoing aortocoronary bypass surgery carries an eightfold risk of future atrial fibrillation and a doubled cardiovascular mortality | EUROPEAN JOURNAL OF CARDIO-THORACIC SURGERY | AHLSSON, A | 2010 | 174 |
| 20 | Fish Oil and Postoperative Atrial Fibrillation The Omega-3 Fatty Acids for Prevention of Post-operative Atrial Fibrillation (OPERA) Randomized Trial | JAMA-JOURNAL OF THE AMERICAN MEDICAL ASSOCIATION | MOZAFFARIAN, D | 2012 | 172 |
| 21 | Obesity and risk of new-onset atrial fibrillation after cardiac surgery | CIRCULATION | HABIB, RH | 2005 | 170 |
| 22 | The gap-junctional protein connexin40 is elevated in patients susceptible to postoperative atrial fibrillation | CIRCULATION | SEVERS, NJ | 2001 | 167 |
| 23 | Statins and postoperative risk of atrial fibrillation following coronary artery bypass grafting | AMERICAN JOURNAL OF CARDIOLOGY | MARIN, F | 2006 | 157 |
| 24 | Competing autonomic mechanisms precede the onset of postoperative atrial fibrillation | JOURNAL OF THE AMERICAN COLLEGE OF CARDIOLOGY | AMAR, D | 2003 | 156 |
| 25 | Vernakalant Hydrochloride for the Rapid Conversion of Atrial Fibrillation After Cardiac Surgery A Randomized, Double-Blind, Placebo-Controlled Trial | CIRCULATION-ARRHYTHMIA AND ELECTROPHYSIOLOGY | KOWEY, PR | 2009 | 152 |
| 26 | Oral amiodarone for prevention of atrial fibrillation after open heart surgery, the Atrial Fibrillation Suppression Trial (AFIST): a randomised placebo-controlled trial | LANCET | KLUGER, J | 2001 | 150 |
| 27 | Association of atrial nicotinamide adenine dinucleotide phosphate oxidase activity with the development of atrial fibrillation after cardiac surgery | JOURNAL OF THE AMERICAN COLLEGE OF CARDIOLOGY | CASADEI, B | 2008 | 148 |
| 28 | Postoperative atrial fibrillation is a major cause of stroke after on-pump coronary artery bypass surgery | ANNALS OF THORACIC SURGERY | BIANCARI, F | 2004 | 145 |
| 29 | Coronary bypass surgery performed off pump does not result in lower in-hospital morbidity than coronary artery bypass grafting performed on pump | CIRCULATION | LEGARE, JF | 2004 | 143 |
| 30 | Omega-3 Fatty Acid Supplementation Does Not Reduce Risk of Atrial Fibrillation After Coronary Artery Bypass Surgery A Randomized, Double-Blind, Placebo-Controlled Clinical Trial | CIRCULATION-ARRHYTHMIA AND ELECTROPHYSIOLOGY | SARAVANAN, P | 2010 | 142 |
| 31 | Left atrial volume predicts the risk of atrial fibrillation after cardiac surgery - A prospective study | JOURNAL OF THE AMERICAN COLLEGE OF CARDIOLOGY | OSRANEK, M | 2006 | 137 |
| 32 | Does Preoperative atrial fibrillation reduce survival after coronary artery bypass grafting? | ANNALS OF THORACIC SURGERY | MCCARTHY, AM | 2004 | 136 |
| 33 | Obesity and metabolic syndrome are independent risk factors for atrial fibrillation after coronary artery bypass graft surgery | CIRCULATION | MATHIEU, P | 2007 | 129 |
| 34 | Clinical prediction rule for atrial fibrillation after coronary artery bypass grafting | JOURNAL OF THE AMERICAN COLLEGE OF CARDIOLOGY | AMAR, D | 2004 | 129 |
| 35 | The persistent problem of new-onset postoperative atrial fibrillation: A single-institution experience over two decades | JOURNAL OF THORACIC AND CARDIOVASCULAR SURGERY | SCHUESSLER, RB | 2011 | 127 |
| 36 | Older age is the strongest predictor of postoperative atrial fibrillation | ANESTHESIOLOGY | AMAR, D | 2002 | 126 |
| 37 | The Society of Thoracic Surgeons 2008 Cardiac Surgery Risk Models: Part 3-Valve Plus Coronary Artery Bypass Grafting Surgery | ANNALS OF THORACIC SURGERY | SHAHIAN, DM | 2009 | 120 |
| 38 | Usefulness of Postoperative Atrial Fibrillation as an Independent Predictor for Worse Early and Late Outcomes After Isolated Coronary Artery Bypass Grafting (Multicenter Australian Study of 19,497 Patients) | AMERICAN JOURNAL OF CARDIOLOGY | SAXENA, A | 2012 | 119 |
| 39 | Red cell transfusion is associated with an increased risk for postoperative atrial fibrillation | ANNALS OF THORACIC SURGERY | KOCH, CG | 2006 | 118 |
| 40 | Human inward rectifier potassium channels in chronic and postoperative atrial fibrillation | CARDIOVASCULAR RESEARCH | DOBREV, D | 2002 | 118 |
| 41 | Additive Costs of Postoperative Complications for Isolated Coronary Artery Bypass Grafting Patients in Virginia | ANNALS OF THORACIC SURGERY | FONNER, E | 2009 | 117 |
| 42 | C-reactive protein is a risk indicator for atrial fibrillation after myocardial revascularization | ANNALS OF THORACIC SURGERY | LO, B | 2005 | 117 |
| 43 | Atrial Myocyte NLRP3/CaMKII Nexus Forms a Substrate for Postoperative Atrial Fibrillation | CIRCULATION RESEARCH | DOBREV, D | 2020 | 116 |
| 44 | N-acetylcysteine for the prevention of postoperative atrial fibrillation: a prospective, randomized, placebo-controlled pilot study | EUROPEAN HEART JOURNAL | OZAYDIN, M | 2008 | 115 |
| 45 | A Randomized Controlled Trial to Prevent Post-Operative Atrial Fibrillation by Antioxidant Reinforcement | JOURNAL OF THE AMERICAN COLLEGE OF CARDIOLOGY | RODRIGO, R | 2013 | 114 |
| 46 | Effects of Angiotensin-Converting Enzyme Inhibitor Therapy on Clinical Outcome in Patients Undergoing Coronary Artery Bypass Grafting | JOURNAL OF THE AMERICAN COLLEGE OF CARDIOLOGY | CAPUTO, M | 2009 | 110 |
| 47 | Relation of inflammatory cytokines to atrial fibrillation after off-pump coronary artery bypass grafting | EUROPEAN JOURNAL OF CARDIO-THORACIC SURGERY | ISHIDA, K | 2006 | 110 |
| 48 | Plasma B-type natriuretic peptide levels predict postoperative atrial fibrillation in patients undergoing cardiac surgery | CIRCULATION | NATALE, A | 2004 | 110 |
| 49 | Dexmedetomidine for reduction of atrial fibrillation and delirium after cardiac surgery (DECADE): a randomised placebo-controlled trial | LANCET | TURAN, A | 2020 | 108 |
| 50 | Bedside Tool for Predicting the Risk of Postoperative Atrial Fibrillation After Cardiac Surgery: The POAF Score | JOURNAL OF THE AMERICAN HEART ASSOCIATION | MARISCALCO, G | 2014 | 108 |
