# Supplementary material for: Bibliometric analysis of postoperative atrial fibrillation following coronary artery bypass grafting surgery
Source: Medicine (Baltimore). 2026 May 15;105(20):e41773. doi: 10.1097/MD.0000000000041773 (PMC13183158; doi:10.1097/MD.0000000000041773)
Supplement: Supplementary file 3 [file medi-105-e41773-s003.docx]

Supplementary Table 3. Titles of top 25 references with the strongest citation bursts

| **Rank** | **References** | **Titles** |
| --- | --- | --- |
| 1 | Aranki SF, 1996, CIRCULATION | Predictors of Atrial Fibrillation After Coronary Artery Surgery |
| 2 | Daoud EG, 1997, NEW ENGL J MED | Preoperative Amiodarone as Prophylaxis against Atrial Fibrillation after Heart Surgery |
| 3 | Mathew JP, 1996, JAMA-J AM MED ASSOC | Atrial fibrillation following coronary artery bypass graft surgery: predictors, outcomes, and resource utilization. MultiCenter Study of Perioperative Ischemia Research Group |
| 4 | Guarnieri T, 1999, J AM COLL CARDIOL | Intravenous amiodarone for the prevention of atrial fibrillation after open heart surgery: the amiodarone reduction in coronary heart (ARCH) trial |
| 5 | Hogue CW, 2000, ANN THORAC SURG | Atrial fibrillation after cardiac operation: risks, mechanisms, and treatment |
| 6 | Giri S, 2001, LANCET | Oral amiodarone for prevention of atrial fibrillation after open heart surgery, the Atrial Fibrillation Suppression Trial (AFIST): a randomised placebo-controlled trial |
| 7 | Zaman AG, 2000, CIRCULATION | Atrial Fibrillation After Coronary Artery Bypass Surgery |
| 8 | Crystal E, 2002, CIRCULATION | Interventions on Prevention of Postoperative Atrial Fibrillation in Patients Undergoing Heart Surgery: A Meta-Analysis |
| 9 | Maisel WH, 2001, ANN INTERN MED | Atrial Fibrillation after Cardiac Surgery |
| 10 | Mathew JP, 2004, JAMA-J AM MED ASSOC | A multicenter risk index for atrial fibrillation after cardiac surgery |
| 11 | Villareal RP, 2004, J AM COLL CARDIOL | Postoperative atrial fibrillation and mortality after coronary artery bypass surgery |
| 12 | Patti G, 2006, CIRCULATION | Randomized Trial of Atorvastatin for Reduction of Postoperative Atrial Fibrillation in Patients Undergoing Cardiac Surgery-Results of the ARMYDA-3 (Atorvastatin for Reduction of MYocardial Dysrhythmia After cardiac surgery) Study |
| 13 | Echahidi N, 2008, J AM COLL CARDIOL | Mechanisms, Prevention, and Treatment of Atrial Fibrillation After Cardiac Surgery |
| 14 | Mariscalco G, 2008, CIRCULATION | Atrial Fibrillation After Isolated Coronary Surgery Affects Late Survival |
| 15 | El-Chami MF, 2010, J AM COLL CARDIOL | New-Onset Atrial Fibrillation Predicts Long-Term Mortality After Coronary Artery Bypass Graft |
| 16 | Maesen B, 2012, EUROPACE | Post-operative atrial fibrillation: a maze of mechanisms |
| 17 | Lapar DJ, 2014, ANN THORAC SURG | Postoperative Atrial Fibrillation Significantly Increases Mortality, Hospital Readmission, and Hospital Costs |
| 18 | Mariscalco G, 2014, J AM HEART ASSOC | Bedside Tool for Predicting the Risk of Postoperative Atrial Fibrillation After Cardiac Surgery: The POAF Score |
| 19 | Kirchhof P, 2016, EUROPACE | 2016 ESC Guidelines for the management of atrial fibrillation developed in collaboration with EACTS |
| 20 | Dobrev D, 2019, NAT REV CARDIOL | Postoperative atrial fibrillation: mechanisms, manifestations and management |
| 21 | Greenberg JW, 2017, EUR J CARDIO-THORAC | Postoperative atrial fibrillation following cardiac surgery: a persistent complication |
| 22 | Filardo G, 2018, HEART | Epidemiology of new-onset atrial fibrillation following coronary artery bypass graft surgery |
| 23 | Lin MH, 2019, STROKE | Perioperative/Postoperative Atrial Fibrillation and Risk of Subsequent Stroke and/or Mortality-A Meta-Analysis |
| 24 | Eikelboom R, 2021, ANN THORAC SURG | Postoperative Atrial Fibrillation After Cardiac Surgery: A Systematic Review and Meta-Analysis |
| 25 | Benedetto U, 2020, CIRCULATION | Postoperative Atrial Fibrillation and Long-Term Risk of Stroke After Isolated Coronary Artery Bypass Graft Surgery |
